# Neuroimmune Crosstalk Between the Peripheral and the Central Immune System in Amyotrophic Lateral Sclerosis

**DOI:** 10.3389/fnagi.2022.890958

**Published:** 2022-05-03

**Authors:** Weiyi Yu, Ji He, Xiying Cai, Zhou Yu, Zhangyu Zou, Dongsheng Fan

**Affiliations:** ^1^Department of Neurology, Peking University Third Hospital, Beijing, China; ^2^Beijing Municipal Key Laboratory of Biomarker and Translational Research in Neurodegenerative Diseases, Beijing, China; ^3^Key Laboratory for Neuroscience, National Health Commission/Ministry of Education, Peking University, Beijing, China; ^4^School of Basic Medical Sciences, Peking University, Beijing, China; ^5^Department of Neurology, Fujian Medical University Union Hospital, Fuzhou, China

**Keywords:** amyotrophic lateral sclerosis, crosstalk, peripheral immunity, CNS barriers, CNS immunity

## Abstract

Amyotrophic lateral sclerosis (ALS) is a fatal disease characterized by the degeneration and death of motor neurons. Systemic neuroinflammation contributes to the pathogenesis of ALS. The proinflammatory milieu depends on the continuous crosstalk between the peripheral immune system (PIS) and central immune system (CIS). Central nervous system (CNS) resident immune cells interact with the peripheral immune cells *via* immune substances. Dysfunctional CNS barriers, including the blood–brain barrier, and blood–spinal cord barrier, accelerate the inflammatory process, leading to a systemic self-destructive cycle. This review focuses on the crosstalk between PIS and CIS in ALS. Firstly, we briefly introduce the cellular compartments of CIS and PIS, respectively, and update some new understanding of changes specifically occurring in ALS. Then, we will review previous studies on the alterations of the CNS barriers, and discuss their crucial role in the crosstalk in ALS. Finally, we will review the moveable compartments of the crosstalk, including cytokines, chemokines, and peripheral immune cells which were found to infiltrate the CNS, highlighting the interaction between PIS and CIS. This review aims to provide new insights into pathogenic mechanisms and innovative therapeutic approaches for ALS.

## Introduction

Amyotrophic lateral sclerosis (ALS) is a neurodegenerative disease typically characterized by adult-onset dysfunction of both upper and lower motor neurons (MNs). The incidence rates of this fatal disease were 1.38 (urban China), 1.5 (United States), 2.08 (Europe) per 100,000 persons (Xu et al., 2020; Burchardt et al., 2022; Mehta et al., 2022), and most patients died within 3–5 years after disease onset (Chia et al., 2018). No clinical therapies have been proven effective except for riluzole and edaravone, which can only delay disease progression (Chen et al., 2016; Scott, 2017; Shefner et al., 2020). The mechanisms underlying ALS pathogenesis are not yet fully understood. ALS is a multifaceted disease, and several mechanisms, including pathogenic gene mutations (Cervantes-Aragón et al., 2020), neuroinflammation (Beers and Appel, 2019), autophagy, mitophagy (French et al., 2018), necrosis (Yuan et al., 2019), aggregation of toxic proteins (Wei et al., 2017), dysfunction of energy metabolism (Vandoorne et al., 2018), and environmental factors (French et al., 2018), have been proven to participate in its pathogenesis.

**FIGURE 1:**
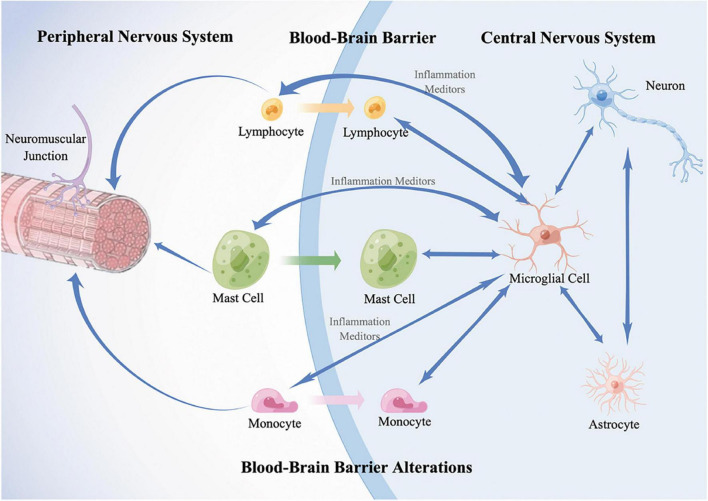
A schematic diagram of immune crosstalk between PNS and CNS. In the CNS, resident immune cells, microglia, are activated and mediate the neuroinflammation by the release of proinflammatory or anti-inflammatory substances such as cytokines and interact with infiltrated peripheral immune cells; astrocytes control the activation, migration, proliferation of microglia. In the PNS, resident immune cells, including T lymphocytes, mast cells, and monocytes are activated and infiltrate along the peripheral motor nerve and neuromuscular junction. Meanwhile, they infiltrate into CNS triggered by microglia-derived inflammation mediators. In addition, dysfunction of CNS barriers, including the blood-brain barrier (BBB) and the blood-spinal cord barrier (BSCB), contribute to the infiltration of peripheral immune cells and accelerate the harmful interaction. As a result, inflammatory responses spread across the two systems contribute to the death of motor neurons (MNs), injuring MN axons, and the dysfunction of neuromuscular junctions. Double-headed arrows represent the communication of two cells. Blue single arrows represent that the cells release inflammatory mediators and influence their targets. Orange, green, and purple arrows represent that the peripheral cells infiltrate into the CNS, respectively.

Accumulating evidence indicates abnormalities in the immune system throughout ALS (Dutta et al., 2020; Theoharides and Tsilioni, 2020). Immune cells are activated and lead to a chronic proinflammatory microenvironment in both the peripheral and central nervous systems in ALS (Masrori et al., 2022). The pro-inflammation in ALS is systemic, and crosstalk exists between the peripheral immune system (PIS) and the central immune system (CIS). To date, crosstalk has not been well defined. With the development of insights into the understanding of ALS, researchers have realized the importance of the continuous interaction and communication of these two systems. CNS resident immune cells and peripheral immune cells interact with each other *via* immune molecules. Dysfunctional CNS barriers, including the blood–brain barrier (BBB) and the blood–spinal cord barrier (BSCB), open the gate for “crosstalk” and are also regulated by the inflammatory environment. As a result, chronic systemic inflammation contributes to the death of MNs, injuring motor neuron axons, and the dysfunction of neuromuscular junctions (Sweeney et al., 2019; Wu Y. et al., 2020; Pan and Nicolazzo, 2022; Figure 1).

## Major Changes of Resident Immune Cells in Amyotrophic Lateral Sclerosis

### Inflammation in Central Nervous System in Amyotrophic Lateral Sclerosis

Inflammation is widespread in the CNS in ALS (Beers and Appel, 2019; Liu et al., 2021). Glial cells, including microglia and astrocytes, trigger neuroinflammatory reactions, interact with infiltrated peripheral immune cells and eventually induce or accelerate neuronal death in CNS in ALS (Cragnolini et al., 2020). Microglia are the resident innate immune cells of the CNS, and mediate the neuroinflammation *via* the release of immune molecules including cytokines and chemokines. Microglia activation is heterogeneous and dependent on the nature of the pathological insult (Mattei and Notter, 2020). Researchers have categorized activated microglia into two opposite types: M1 (toxic or proinflammatory) or M2 (neuroprotective or anti-proinflammatory) microglia (Guo et al., 2022). However, researchers have recently realized that there is a continuum of phenotypes between M1 and M2 in ALS (Li et al., 2019), such as disease-associated microglia (DAM) (Krasemann et al., 2017; Dols-Icardo et al., 2020) and receptor-interacting protein kinase 1 (RIPK1)—regulated inflammatory microglia (RRIMs) (Mifflin et al., 2021). In general, accumulating studies have proven that microglia show an anti-inflammatory phenotype and protect MNs at the onset of the disease, while end-stage microglia shift to a proinflammatory phenotype and aggravate the neurodegeneration of MNs in ALS (Liu et al., 2021; Masrori et al., 2022). Astrocytes are the most common glial cells in the brain, maintain the CNS barriers (Signorile et al., 2021), secrete neurotrophic and neuroprotective factors, regulate neurotransmitter uptake and recycling, and promote neurogenesis (Gharbi et al., 2020). Studies have identified a role for astrocytes as immune modulators, as they may control the activation, migration, and proliferation of microglia (Sunnemark et al., 2005; Ouali et al., 2018).

### Immune Activation in the Periphery in Amyotrophic Lateral Sclerosis

Peripheral immune abnormalities exist in ALS (McCombe et al., 2020). In general, the chronic peripheral immune response is proinflammatory in ALS. Lymphocytes, monocytes (including macrophages), neutrophils, natural killer (NK) cells, and mast cells (MCs) are peripheral resident immune cells. ALS patients were found to have elevated total leukocyte counts in blood (Murdock et al., 2017). In peripheral blood, most studies suggest decreased levels of neuroprotective CD4 T lymphocytes while the subgroup of CD4 T lymphocytes, regulatory T cells (Tregs), are reduced and dysfunctional in ALS patients. In ALS, the number of cytotoxic CD8 T lymphocytes in peripheral blood is controversial. NK T lymphocytes are thought to be harmful in ALS and are increased in peripheral blood in patients with ALS (Finkelstein et al., 2011; Perner et al., 2018; Giovannelli et al., 2020; Nishihara et al., 2020; Rolfes et al., 2021). B lymphocytes are merely discussed in ALS and studies suggest that they play a supplementary role in the pathogenesis of ALS (Naor et al., 2009; Pennati et al., 2018). Alterations in the proportion of monocytes were reported and circulating monocytes from ALS patients preferentially differentiated to a proinflammatory phenotype (Liu et al., 2016; Du et al., 2020). The numbers of neutrophils are increased in the peripheral blood and show a significant correlation with disease progression (Murdock et al., 2017; Leone et al., 2022). NK cells are innate immune cells and mediate cytotoxicity. Levels of NK cells in the blood of ALS patients are increased and could be pathogenic (Gustafson et al., 2017; Murdock et al., 2017). An increased number of circulating MCs was shown in ALS mice while there was a lack of evidence in ALS patients (Trias et al., 2018; Harcha et al., 2021).

Distal axonopathy is a recognized pathological feature of ALS (Nardo et al., 2016). Recruitment of activated MCs, macrophages, and neutrophils along the degenerating motor axons in sciatic nerves and skeletal muscle is observed in ALS (Chiu et al., 2009; Angelini et al., 2020; Trias et al., 2020). Peripheral immune cells can also infiltrate into CNS and exert an effect on motor neurons and glial cells, which will be discussed below. Peripheral immune cells have been increasingly discussed in their prognostic role. In this regard, with the development of technology and understanding, researchers have turned to exploring a specific population or a single myeloid subpopulation to categorize or monitor patients (Murdock et al., 2017; Leone et al., 2022).

## Alteration of the Central Nervous System Barriers in Amyotrophic Lateral Sclerosis

CNS barriers are formed by a layer of endothelial cells, connected by inter endothelial tight junctions (TJs), adhesion proteins, and cytoplasm (Bull et al., 2022). A basement membrane called the basal lamina (BL) ensheathed by pericytes and astrocytic end-feet supports endothelial cells and associated pericytes (Lochhead et al., 2020; Yu et al., 2020). They make up the physical barriers of the CNS while the biochemical barriers of CNS are imparted by various transport systems. Alterations in brain barriers have been observed in the early stage in ALS patients and mice, suggesting that the impairment may contribute to the pathogenesis (Bull et al., 2022). The alterations are summarized as follows: disruption of the integrity of physical barriers, function modulation of biochemical barriers, and secretion of neuroimmune-related substances by barrier cells in the immune response (Erickson and Banks, 2018; Kakaroubas et al., 2019; Gil-Martins et al., 2020). CNS barriers act as the center point in humoral-based communications between the CIS and PIS. A better understanding of how the integrity or function of CNS barriers is altered may provide approaches to terminate the harmful crosstalk in ALS.

### Disruption of the Integrity of Physical Barriers in Amyotrophic Lateral Sclerosis

Multiple studies have found alterations of the ultrastructure of CNS barriers in ALS patients, including swelling and cytoplasmic vacuolization of microvascular endothelial cells, reduced pericyte coverage, and detachment of astrocyte end-feet processes from endothelial cells in the spinal cord of ALS patients (Miyazaki et al., 2011; Garbuzova-Davis et al., 2012; Yamadera et al., 2015). Ultrastructural alterations have also been observed in the brain stem and cervical and lumbar spinal cords, but not in the motor cortex of ALS mice. The alterations have been noted to occur at the early disease stage and worsen with disease progression (Garbuzova-Davis et al., 2012; Winkler et al., 2013). TJs are formed by multiple proteins, such as zonula occludens-1 (ZO-1) and occludin, and prevent the paracellular movement of solutes (Winkler et al., 2013). A significant reduction in the expression of TJs and adhesion proteins such as ZO-1 and occludin was observed in the spinal cord of both ALS patients and mice (Pan and Nicolazzo, 2022). Despite the change in adhesion proteins, the morphological structures of TJs were found to be well preserved under electron microscopy in the spinal cord of postmortem ALS patients (Sasaki, 2015). Although morphological structures of TJs are preserved, the detection of endogenous proteins in the CNS suggests the increased paracellular permeability and leakiness of CNS barriers (Waters et al., 2021). Furthermore, BL thickening is observed in both ALS patients and mice. The detachment of endothelial cells exposes BL to plasma proteins, fibrin, and collagen IV within the BL, which then accumulate, leading to BL thickening (Sasaki, 2015). As BL abnormalities are detected in the early stage of ALS mice, these findings suggest that it may occur as a compensatory mechanism or a reparative process (Nguyen et al., 2021). Based on these findings, ultrastructural abnormalities and reduced expression of TJs adhesion proteins may contribute to compromised junctional integrity and an increase in paracellular permeability, permitting peripheral substances and cell access to the CNS. Therefore, it improves the communication of PIS and CIS, and accelerates the systemic proinflammation.

### Functional Modulation of the Biochemical Central Nervous System Barriers

Biochemical CNS barriers are imparted by various transport systems, such as ATP-binding cassette (ABC) protein. They can effectively exclude various endogenous and exogenous toxins from the endothelial cells to maintain cellular homeostasis. The most well-studied ABC protein, P-glycoprotein (P-gp), is a major efflux transporter for small, lipid-soluble molecules expressed on CNS barriers (Gil-Martins et al., 2020). The expression and activity of P-gp are upregulated in both ALS patients and mice (Jablonski et al., 2012; Qosa et al., 2016; Chan et al., 2017; van Vliet et al., 2020). Tumor necrosis factor α (TNF-α) and growth factor-beta 1(TGF-β1) were shown to upregulate the expression and activity of P-gp in mice and rats (Dohgu et al., 2004; Bauer et al., 2007). As levels of TNF-α and TGF-β1 are increased in ALS patients and mice (Bougea, 2019; Tortelli et al., 2020), they are associated with the overexpression of P-gp. Moreover, astrocytes are also suspected to be responsible for the increased expression of P-gp in ALS dependent on ALS genotypes. For example, cocultured ALS-associated-mutant SOD1 astrocytes impacted P-gp in nearby endothelial cells by secreting soluble factors such as TNF-α, chemokines, and reactive oxygen species (ROS) (Ji et al., 2013). Meanwhile, ALS-associated mutant C9orf72 astrocytes have been shown to have no effects on endothelial P-gp expression (Mohamed et al., 2019). Additionally, the expression of breast cancer resistance protein (BRCP), another efflux transporter, is upregulated in ALS patients and mice (Jablonski et al., 2012; Chan et al., 2017; van Vliet et al., 2020). In general, the increased P-gp and BRCP abundance and activities at the CNS barriers suggest the modulation of interface functions of biochemical CNS barriers, which may ultimately influence the development of ALS.

### Barrier Cells Secrete Neuroimmune-Related Substances in the Immune Response

Barrier cells, including endothelial cells, pericytes, and astrocytes, secrete neuroimmune-related substances in response to immune stimulation from peripheral or central immune cells. Brain endothelial cells (BECs) can constitutively secrete interleukin 6 (IL-6), prostaglandins, and nitric oxide in response to different stimuli (Iannucci et al., 2020; Charoensaensuk et al., 2021). As the number of pericytes is reduced in ALS (Winkler et al., 2013), its inflammatory-mediated role may also contribute to ALS pathologies. Compared to other barrier cells, pericytes are the most sensitive to TNF-α and can release IL-6 and macrophage inflammatory protein-1α (MIP-1α, also known as CCL3) in response (Matsumoto et al., 2014). Inflammatory reactive pericytes support neutrophil transmigration by the release of IL-8 and matrix metalloproteinase-9 (MMP-9), leading to the subsequent development of neuroinflammation (Pieper et al., 2013). Astrocytes are activated in the immune response in ALS. On the one hand, astrocytes control the activation, migration, and proliferation of microglia *via* multiple inflammatory factors, and secrete proteins such as MCP-1 which mediates monocyte migration to amplify neuroinflammation in the CNS (Ouali et al., 2018; Izrael et al., 2020). On the other hand, biochemical substances such as nitric oxide, vascular endothelial growth factors (VEGF), glial cell line-derived neurotrophic factor (GDNF), and MM-9 released from reactive astrocytes on barriers regulate the expression of TJ proteins and the proliferation of endothelial cells, thus influencing the integrity and permeability of CNS barriers (Spiller et al., 2019; Izrael et al., 2020; Takata et al., 2021; Qin et al., 2022). Therefore, barrier cells can not only transfer information from one side to the other side (such as PIS to CIS) but are also involved in mediating the inflammatory microenvironment.

## The Crosstalk From the Peripheral Immune System to the Central Nervous System Contributes to the Systemic Inflammatory Milieu of Amyotrophic Lateral Sclerosis

In ALS, injured MNs interact with glia, and they release certain levels of cytokines and chemokines, followed by the recruitment of innate and adaptive immune cells to infiltrate the CNS to promote inflammation. Proinflammatory signaling spreads from CIS to PIS and from PIS to CIS, thereby contributing to the systemic inflammatory milieu of ALS.

### Cytokines and Chemokines in Amyotrophic Lateral Sclerosis

Many cytokines and chemokines, such as IL-1, IL-6, TNF, and CC chemokine ligand 2 (CCL2), have been shown to cross CNS barriers while the barriers mediate their transport, penetration, and uptake (Zhao et al., 2020; Bull et al., 2022). On the one hand, due to the activation of immune cells, the levels of cytokines and chemokines are significantly changed in ALS (Sun et al., 2022). Their major roles in PIS or CIS in ALS are summarized in Table 1. On the other hand, elevated levels of proinflammatory mediators increase the permeability of the CNS barriers, act directly on their receptors to alter the function of resident cells, induce immune cell trafficking, and exacerbate barrier disruption and neuroinflammation (Wang et al., 2014; Banks, 2015; Erickson et al., 2020).

**TABLE 1:** The major role of cytokines and chemokines in ALS.

Molecules	Secreting cells	Change in ALS	Role in the immune system	References
TNF-α	Macrophages, T lymphocytes, NK cells	Increased	Proinflammation: activation of immune cells	Tortarolo et al., 2017; Bougea, 2019
IL-1β	Monocytes, macrophages; M1 microglia	Increased	Proinflammation: activation of immune cells	Italiani et al., 2014; Sun et al., 2022
IL-6	Immune cells, endothelial cells, myocytes	Increased/unchanged	Proinflammation: activation of immune cells	Martinez-Merino et al., 2018; Pronto-Laborinho et al., 2019; Wosiski-Kuhn et al., 2019
IL-8/CXCL8	Monocytes, endothelial cells	Increased	Proinflammation: recruitment of neutrophils, activation of glial cells	Rusconi et al., 2017
IL-10	Monocytes, T lymphocytes, and B lymphocytes; immunosuppressive microglia (M2)	Increased/increased in the early stage and decreased during disease progression	Anti-inflammation: limiting excessive production of proinflammatory cytokines, ROS.	Batista et al., 2009; Noh et al., 2014; Strickland et al., 2020
IL-13	Th2 cells, CD4 cells, natural killer T cells, mast cells, basophils, eosinophils, and neurocytes	Increased	Controversial mechanism: proinflammation: enhancing MCP-1 expression in monocytes and macrophages; anti-inflammation: induce infiltration to the injured spinal cord and anti-inflammatory polarity of macrophages	Shi et al., 2007; Lu et al., 2016; Amo-Aparicio et al., 2021
IL-17a	Th17 cells, CD8^+^ T cells, mast cells; astrocytes	Increased	Proinflammation	Fiala et al., 2010; Jin et al., 2021
IL-33	Multiple cells	Induced	Anti-inflammation: decreasing the proportion of CD4^+^ and CD8^+^ T cell populations, regulating mast cells function	Lin et al., 2012; Korhonen et al., 2019
G-CSF	Monocytes and macrophages	Induced	Dual mechanism: inducing mobilization of bone marrow cells from bone to the peripheral, stimulating proliferation, inducing the recruitment of microglia in the damaged areas	Salamone et al., 2020
CXCL13	MNs	Increased	Anti-inflammation	Trolese et al., 2020
CXCL12	Bone marrow stromal cells	Increased	Proinflammation: development of T and B lymphocytes, influencing survival of mature Lymphocytes, microglial pathology, and permeability of CNS barriers	Li and Ransohoff, 2008; Rabinovich-Nikitin et al., 2016
CX3CL1	MNs, microglia	Increased	Proinflammation: activation of microglia	Zhang et al., 2018
CCL2	MNs, microglia, astrocytes	Increased	Proinflammation: activation and recruitment of NK cells, T cells	Garofalo et al., 2020
CCL5	T lymphocytes, macrophages, endothelial cells	Increased	Proinflammation: proliferation and activation of T lymphocytes, monocytes	Perner et al., 2018
CCL18/MIP-4	DC	No changed	Proinflammation: attracting lymphocytes toward DC and activated macrophages, activation of microglia	Martinez-Merino et al., 2018

*TNF-α, Tumor Necrosis Factor; IL, Interleukin; G-CSF, Recombinant Human Granulocyte-Colony Stimulating Factor; CCL, C-C Motif Ligand; CX3CL1, C-X3-C Motif Chemokine Ligand 1; CXCL, C-X-C Motif Chemokine Ligand; MIP-4, Macrophage Inflammatory Protein-4; DC, Dendritic Cell.*

### Central Nervous System Infiltration of Peripheral Immune Cells in Amyotrophic Lateral Sclerosis

Increasing evidence shows that many peripheral leukocytes are first activated in PIS and then migrate into the CNS in ALS (Angelini et al., 2020). The regulation of leukocyte trafficking to the CNS is multifaceted and depends on the activation state of the leukocytes, TJ complexes at the endothelial interface, and the inflammatory microenvironment in the CNS and PNS (Congdon et al., 2019; Trolese et al., 2020; Marchetti et al., 2022). As peripheral leukocytes can be easily monitored, and intrathecal or intracerebroventricular is associated with several risks, targeting peripheral leukocytes may be feasible in ALS treatment. Therefore, a better understanding of how peripheral immune cells infiltrate into the CNS is needed.

#### T Lymphocytes

The infiltration of T lymphocytes in ALS is well-known (Rolfes et al., 2021). Chemokines and chemokine receptors are critical for parenchymal infiltration. The chronic inflammatory milieu induces the upregulation of leukocyte cell adhesion on the surface of endothelial cells, which binds to CD6 expressed on T lymphocytes, allowing their entry into the brain parenchyma (Larochelle et al., 2012). In addition, T lymphocyte-derived TNF-α and IL-17 induce the secretion of MM-9 in immune cells and MNs, facilitating T lymphocyte infiltration into the CNS (Song et al., 2015). A large amount of evidence highlights the differences between T-cell subsets and their specific mechanisms of entry into the CNS in ALS. For example, endothelial cells secrete chemokines such as CXCL9, CXCL10, CXCL11, CCL19, CCL21, and MCP-1 to recruit CD4^+^ T cells through CNS barriers. Treg cells, which have an inhibitory effect on neuroinflammation, are activated and recruited to the CNS *via* CCL5/CCR5 and CCL6/CCR6 mechanisms to inhibit the activation of microglia in the early phase of the disease (Zhao et al., 2012; Beers et al., 2017). CD8^+^ T cells show intense infiltration and induce MN death *via* MHC-I expressed in activated microglia and injured MNs (Coque et al., 2019; Liu et al., 2020).

#### Mast Cells

Findings in previous studies suggested that MCs play a role in early degeneration in the PNS and have a ripple effect on neuronal damage (Trias et al., 2018; Angelini et al., 2020). Later studies confirmed the infiltration of MCs in the spinal cord of ALS patients (Fiala et al., 2010; Kovacs et al., 2021). The expression of receptors on MCs is affected by IL-6, CCL5, and TNF-α released by activated microglia, resulting in the regulation of MC activation and CNS recruitment (Jones et al., 2019). Moreover, MCs can release proteases to TJs and extracellular matrix components, thus influencing the permeability and integrity of the BBB and leading to CNS invasion of MCs (Mattila et al., 2011; Jones et al., 2019).

#### Monocytes

Limited numbers of activated peripheral monocytes infiltrate the CNS and influence neuroinflammation in ALS (Chiot et al., 2020). Previous studies indicate an alteration in the proportion of monocytes in ALS (Mcgill et al., 2021). In patients with rapidly progressing ALS, monocytes in the peripheral circulation are usually in a proinflammatory state (Zhao et al., 2017). Recently, peripheral monocytes have been proven to infiltrate the CNS, which is related to improved motoneuron survival in ALS, but infiltration may be limited (Peake et al., 2017). In addition, monocyte-derived macrophages are activated in ALS. Activated macrophages exert neuroprotective functions by misfolding protein clearance during the disease (Chiu et al., 2009; Shiraishi et al., 2021). Macrophages also showed limited infiltration to the CNS. The evidence may suggest that the accumulating monocytes in the CNS were due to the proliferation of infiltrated cells instead of the infiltration of accumulated circulated monocytes (Chiot et al., 2020).

#### Other Immune Cells: Neutrophils, Natural Killer Cells

Few studies have discussed the role of neutrophils and NK cells in neuroimmune crosstalk. However, considering that there is a significant correlation between an increase in the number of neutrophils and NK cells in the peripheral blood and disease progression (Murdock et al., 2017; Leone et al., 2022), and their role in innate immune responses, it is believed to affect neuroinflammation of the CNS in complicated ways. For example, end-stage ALS mice showed a high NK cell frequency in the spinal cord (Finkelstein et al., 2011). NK cell-derived IFN-γ induces microglia toward an inflammatory phenotype, regulates the release of CCL2, a chemokine that can regulate CNS infiltration, from MNs, and impairs Treg cell migration (Garofalo et al., 2020). More studies are needed.

## Conclusion

Previous investigations of neuroinflammation in ALS have mainly focused on the relationship between the two immune systems and ALS, respectively. Nevertheless, much less is discussed on the crosstalk between PIS and CIS in ALS, especially the role of the CNS barriers. In this review, we updated the understanding of the relationship between neuroinflammation and ALS. Crosstalk involving central immune cells and peripheral immune cells, CNS barriers, cytokines and chemokines was fully discussed. The dysfunction of all these elements contributed to the non-cellulous death of MNs. Crosstalk plays an important role in the systemic inflammatory milieu in ALS. It should be fully considered for mechanisms and treatment discovery in ALS.

CNS barriers play a crucial role in the crosstalk; thus, they may be a target when optimizing medicine use with ALS. For example, riluzole is a substrate for P-gp and BRCP expressed on CNS barriers so the drug efficacy may be negatively affected (Jablonski et al., 2014). Inhibitors of P-gp have been proven to improve drug delivery in ALS mice (Gil-Martins et al., 2020), but clinical trials should be conducted and more investigations are needed. In addition, a combination of possible CNS barriers- impaired medicine with other therapies may be beneficial. Angiopoietin-1 promotes angiogenesis in the CNS and reduces vascular permeability. The C16 peptide repairs vessels and inhibit transmigration and infiltration of leukocytes without the side effect of systemic immunosuppression. The roles of these two medicines have been well studied in animal models of CNS inflammation (Wu D. et al., 2020), but further experiments in ALS are needed. Notably, the effect of neuroinflammation is dual, as it exerts a neurotoxic or neuroprotective effect during the disease. In conclusion, normalizing immune crosstalk and homeostasis instead of suppressing inflammation may provide a potential therapeutic target and direction for future study.

## Author Contributions

WY and JH wrote the manuscript and reviewed the literature under the supervision of DF. XC and ZY drafted the figure and table. ZZ revised the manuscript. All authors contributed to the article and approved the submitted version.

## Conflict of Interest

The authors declare that the research was conducted in the absence of any commercial or financial relationships that could be construed as a potential conflict of interest.

## Publisher’s Note

All claims expressed in this article are solely those of the authors and do not necessarily represent those of their affiliated organizations, or those of the publisher, the editors and the reviewers. Any product that may be evaluated in this article, or claim that may be made by its manufacturer, is not guaranteed or endorsed by the publisher.

## References

[B1] Amo-Aparicio J. Garcia-Garcia J. Francos-Quijorna I. Urpi A. Esteve-Codina A. Gut M. (2021). Interleukin-4 and interleukin-13 induce different metabolic profiles in microglia and macrophages that relate with divergent outcomes after spinal cord injury. *Theranostics* 11 9805–9820. 10.7150/thno.65203 34815787 PMC8581417

[B2] Angelini D. F. De Angelis F. Vacca V. Piras E. Parisi C. Nutini M. (2020). Very early involvement of innate immunity in peripheral nerve degeneration in SOD1-G93A mice. *Front. Immunol.* 11:575792. 10.3389/fimmu.2020.575792 33329541 PMC7714949

[B3] Banks W. A. (2015). The blood-brain barrier in neuroimmunology: tales of separation and assimilation. *Brain Behav. Immunity* 44 1–8. 10.1016/j.bbi.2014.08.007 25172555 PMC4275374

[B4] Batista M. J. Lopes R. D. Seelaender M. C. Lopes A. C. (2009). Anti-inflammatory effect of physical training in heart failure: role of TNF-alpha and IL-10. *Arq. Bras. Cardiol.* 93 692–700. 20379646

[B5] Bauer B. Hartz A. M. Miller D. S. (2007). Tumor necrosis factor alpha and endothelin-1 increase P-glycoprotein expression and transport activity at the blood-brain barrier. *Mol. Pharmacol.* 71 667–675. 10.1124/mol.106.029512 17132686

[B6] Beers D. R. Appel S. H. (2019). Immune dysregulation in amyotrophic lateral sclerosis: mechanisms and emerging therapies. *Lancet Neurol.* 18 211–220. 10.1016/S1474-4422(18)30394-6 30663610

[B7] Beers D. R. Zhao W. Wang J. Zhang X. Wen S. Neal D. (2017). ALS patients’ regulatory T lymphocytes are dysfunctional, and correlate with disease progression rate and severity. *JCI Insight* 2:e89530. 10.1172/jci.insight.89530 28289705 PMC5333967

[B8] Bougea A. (2019). Is TNF inhibitor exposure a risk factor for amyotrophic lateral sclerosis? *Fund. Clin. Pharmacol.* 33 687–688. 10.1111/fcp.12513 31618791

[B9] Bull D. Schweitzer C. Bichsel C. Britschgi M. Gutbier S. (2022). Generation of an hiPSC-Derived Co-Culture system to assess the effects of neuroinflammation on blood–brain barrier integrity. *Cells* 11:419. 10.3390/cells11030419 35159229 PMC8834542

[B10] Burchardt J. M. Mei X. W. Ranger T. McDermott C. J. Radunovic A. Coupland C. (2022). Analysis of incidence of motor neuron disease in England 1998-2019: Use of three linked datasets. *Amyotroph. Lateral Scler. Frontotemporal Degener.* [E-pub ahead of print]. 10.1080/21678421.2021.2016837 35103515 PMC9344929

[B11] Cervantes-Aragón I. Ramírez-García S. A. Baltazar-Rodríguez L. M. García-Cruz D. Castañeda-Cisneros G. (2020). Genetic approach in amyotrophic lateral sclerosis. *Gaceta Mexico* 155 475–482. 10.24875/GMM.M20000335 32091028

[B12] Chan G. N. Y. Evans R. A. Banks D. B. Mesev E. V. Miller D. S. Cannon R. E. (2017). Selective induction of P-glycoprotein at the CNS barriers during symptomatic stage of an ALS animal model. *Neurosci. Lett.* 639 103–113. 10.1016/j.neulet.2016.12.049 28011392 PMC5278641

[B13] Charoensaensuk V. Chen Y. Lin Y. Ou K. Yang L. Lu D. (2021). Porphyromonas gingivalis induces proinflammatory cytokine expression leading to apoptotic death through the oxidative Stress/NF-κB pathway in brain endothelial cells. *Cells* 10:3033. 10.3390/cells10113033 34831265 PMC8616253

[B14] Chen L. Liu X. Tang L. Zhang N. Fan D. (2016). Long-Term use of riluzole could improve the prognosis of sporadic amyotrophic lateral sclerosis patients: a Real-World cohort study in china. *Front. Aging Neurosci.* 8:246. 10.3389/fnagi.2016.00246 27822184 PMC5075535

[B15] Chia R. Chiò A. Traynor B. J. (2018). Novel genes associated with amyotrophic lateral sclerosis: diagnostic and clinical implications. *Lancet Neurol*. 17 94–102. 10.1016/S1474-4422(17)30401-5 29154141 PMC5901717

[B16] Chiot A. Zaïdi S. Iltis C. Ribon M. Berriat F. Schiaffino L. (2020). Modifying macrophages at the periphery has the capacity to change microglial reactivity and to extend ALS survival. *Nat. Neurosci.* 23 1339–1351. 10.1038/s41593-020-00718-z 33077946

[B17] Chiu I. M. Phatnani H. Kuligowski M. Tapia J. C. Carrasco M. A. Zhang M. (2009). Activation of innate and humoral immunity in the peripheral nervous system of ALS transgenic mice. *Proc. Natl. Acad. Sci*. 106 20960–20965. 10.1073/pnas.0911405106 19933335 PMC2791631

[B18] Congdon K. L. Sanchez-Perez L. A. Sampson J. H. (2019). Effective effectors: how T cells access and infiltrate the central nervous system. *Pharmacol. Therapeut.* 197 52–60. 10.1016/j.pharmthera.2018.12.007 30557632 PMC7164682

[B19] Coque E. Salsac C. Espinosa-Carrasco G. Varga B. Degauque N. Cadoux M. (2019). Cytotoxic CD8+ T lymphocytes expressing ALS-causing SOD1 mutant selectively trigger death of spinal motoneurons. *Proc. Natl. Acad. Sci*. 116 2312–2317. 10.1073/pnas.1815961116 30674678 PMC6369778

[B20] Cragnolini A. Lampitella G. Virtuoso A. Viscovo I. Panetsos F. Papa M. (2020). Regional brain susceptibility to neurodegeneration: What is the role of glial cells? *Neural Regen. Res.* 15 838–842. 10.4103/1673-5374.268897 31719244 PMC6990768

[B21] Dohgu S. Yamauchi A. Takata F. Naito M. Tsuruo T. Higuchi S. (2004). Transforming growth factor-beta1 upregulates the tight junction and P-glycoprotein of brain microvascular endothelial cells. *Cell Mol. Neurobiol.* 24 491–497. 10.1023/b:cemn.0000022776.47302.ce 15206827 PMC11529953

[B22] Dols-Icardo O. Montal V. Sirisi S. López-Pernas G. Cervera-Carles L. Querol-Vilaseca M. (2020). Motor cortex transcriptome reveals microglial key events in amyotrophic lateral sclerosis. *Neurol. Neuroimmunol. Neuroinflamm*. 7:e829. 10.1212/NXI.0000000000000829 32669313 PMC7371375

[B23] Du Y. Zhao W. Thonhoff J. R. Wang J. Wen S. Appel S. H. (2020). Increased activation ability of monocytes from ALS patients. *Exp. Neurol.* 328:113259. 10.1016/j.expneurol.2020.113259 32105709

[B24] Dutta K. Thammisetty S. S. Boutej H. Bareil C. Julien J. (2020). Mitigation of ALS pathology by Neuron-Specific inhibition of nuclear factor kappa b signaling. *J. Neurosci*. 40 5137–5154. 10.1523/JNEUROSCI.0536-20.2020 32457070 PMC7314413

[B25] Erickson M. A. Banks W. A. (2018). Neuroimmune axes of the blood–brain barriers and blood–brain interfaces: bases for physiological regulation, disease states, and pharmacological interventions. *Pharmacol. Rev.* 70 278–314. 10.1124/pr.117.014647 29496890 PMC5833009

[B26] Erickson M. A. Wilson M. L. Banks W. A. (2020). In vitro modeling of blood–brain barrier and interface functions in neuroimmune communication. *Fluids Barriers CNS* 17:26. 10.1186/s12987-020-00187-3 32228633 PMC7106666

[B27] Fiala M. Chattopadhay M. La Cava A. Tse E. Liu G. Lourenco E. (2010). IL-17A is increased in the serum and in spinal cord CD8 and mast cells of ALS patients. *J. Neuroinflammation*. 7:76. 10.1186/1742-2094-7-76 21062492 PMC2992053

[B28] Finkelstein A. Kunis G. Seksenyan A. Ronen A. Berkutzki T. Azoulay D. (2011). Abnormal changes in NKT cells, the IGF-1 axis, and liver pathology in an animal model of ALS. *PLoS One* 6:e22374. 10.1371/journal.pone.0022374 21829620 PMC3149057

[B29] French P. W. Ludowyke R. Guillemin G. J. (2018). Fungal neurotoxins and sporadic amyotrophic lateral sclerosis. *Neurotox. Res.* 35 969–980. 10.1007/s12640-018-9980-5 30515715

[B30] Garbuzova-Davis S. Hernandez-Ontiveros D. G. Rodrigues M. C. O. Haller E. Frisina-Deyo A. Mirtyl S. (2012). Impaired blood–brain/spinal cord barrier in ALS patients. *Brain Res.* 1469 114–128. 10.1016/j.brainres.2012.05.056 22750125

[B31] Garofalo S. Cocozza G. Porzia A. Inghilleri M. Raspa M. Scavizzi F. (2020). Natural killer cells modulate motor neuron-immune cell cross talk in models of Amyotrophic Lateral Sclerosis. *Nat. Commun.* 11:1773. 10.1038/s41467-020-15644-8 32286313 PMC7156729

[B32] Gharbi T. Zhang Z. Yang G. (2020). The function of astrocyte mediated extracellular vesicles in central nervous system diseases. *Front. Cell Dev. Biol.* 8:568889. 10.3389/fcell.2020.568889 33178687 PMC7593543

[B33] Gil-Martins E. Barbosa D. J. Silva V. Remião F. Silva R. (2020). Dysfunction of ABC transporters at the blood-brain barrier: role in neurological disorders. *Pharmacol. Therapeut.* 213:107554. 10.1016/j.pharmthera.2020.107554 32320731

[B34] Giovannelli I. Heath P. Shaw P. J. Kirby J. (2020). The involvement of regulatory T cells in amyotrophic lateral sclerosis and their therapeutic potential. *Amyotroph. Lateral Scler. Frontotemporal Degener*. 21 435–444. 10.1080/21678421.2020.1752246 32484719

[B35] Guo S. Wang H. Yin Y. (2022). Microglia polarization from m1 to m2 in neurodegenerative diseases. *Front. Aging Neurosci.* 14:815347. 10.3389/fnagi.2022.815347 35250543 PMC8888930

[B36] Gustafson M. P. Staff N. P. Bornschlegl S. Butler G. W. Maas M. L. Kazamel M. (2017). Comprehensive immune profiling reveals substantial immune system alterations in a subset of patients with amyotrophic lateral sclerosis. *PLoS One* 12:e182002. 10.1371/journal.pone.0182002 28742871 PMC5526569

[B37] Harcha P. A. Garcés P. Arredondo C. Fernández G. Sáez J. C. van Zundert B. (2021). Mast cell and astrocyte hemichannels and their role in Alzheimer’s disease, ALS, and harmful stress conditions. *Int. J. Mol. Sci.* 22:1924. 10.3390/ijms22041924 33672031 PMC7919494

[B38] Iannucci J. Rao H. V. Grammas P. (2020). High glucose and Hypoxia-Mediated damage to human brain microvessel endothelial cells induces an altered, Pro-Inflammatory phenotype in BV-2 microglia in vitro. *Cell. Mol. Neurobiol.* 42 985–996. 10.1007/s10571-020-00987-z 33136275 PMC8942976

[B39] Italiani P. Carlesi C. Giungato P. Puxeddu I. Borroni B. Bossu P. (2014). Evaluating the levels of interleukin-1 family cytokines in sporadic amyotrophic lateral sclerosis. *J. Neuroinflamm*. 11:94. 10.1186/1742-2094-11-94 24884937 PMC4039322

[B40] Izrael M. Slutsky S. G. Revel M. (2020). Rising stars: astrocytes as a therapeutic target for ALS disease. *Front. Neurosci.* 14:824. 10.3389/fnins.2020.00824 32848579 PMC7399224

[B41] Jablonski M. R. Jacob D. A. Campos C. Miller D. S. Maragakis N. J. Pasinelli P. (2012). Selective increase of two ABC drug efflux transporters at the blood–spinal cord barrier suggests induced pharmacoresistance in ALS. *Neurobiol. Dis.* 47 194–200. 10.1016/j.nbd.2012.03.040 22521463 PMC3367047

[B42] Jablonski M. R. Markandaiah S. S. Jacob D. Meng N. J. Li K. Gennaro V. (2014). Inhibiting drug efflux transporters improves efficacy of ALS therapeutics. *Ann. Clin. Transl. Neurol*. 1 996–1005. 10.1002/acn3.141 25574474 PMC4284125

[B43] Ji B. S. Cen J. He L. Liu M. Liu Y. Q. Liu L. (2013). Modulation of P-glycoprotein in rat brain microvessel endothelial cells under oxygen glucose deprivation. *J. Pharm. Pharmacol.* 65 1508–1517. 10.1111/jphp.12122 24028618

[B44] Jin M. Akgün K. Ziemssen T. Kipp M. Günther R. Hermann A. (2021). Interleukin-17 and th17 lymphocytes directly impair motoneuron survival of wildtype and FUS-ALS mutant human iPSCs. *Int. J. Mol. Sci.* 22:8042. 10.3390/ijms22158042 34360808 PMC8348495

[B45] Jones M. K. Nair A. Gupta M. (2019). Mast cells in neurodegenerative disease. *Front. Cell. Neurosci.* 13:171. 10.3389/fncel.2019.00171 31133804 PMC6524694

[B46] Kakaroubas N. Brennan S. Keon M. Saksena N. K. (2019). Pathomechanisms of Blood-Brain barrier disruption in ALS. *Neurosci. J*. 2019:2537698. 10.1155/2019/2537698 31380411 PMC6652091

[B47] Korhonen P. Pollari E. Kanninen K. M. Savchenko E. Lehtonen Š Wojciechowski S. (2019). Long-term interleukin-33 treatment delays disease onset and alleviates astrocytic activation in a transgenic mouse model of amyotrophic lateral sclerosis. *IBRO Rep.* 6 74–86. 10.1016/j.ibror.2019.01.005 30705990 PMC6348738

[B48] Kovacs M. Alamón C. Maciel C. Varela V. Ibarburu S. Tarragó L. (2021). The pathogenic role of c-Kit+ mast cells in the spinal motor neuron-vascular niche in ALS. *Acta Neuropathol. Commun.* 9:136. 10.1186/s40478-021-01241-3 34389060 PMC8361844

[B49] Krasemann S. Madore C. Cialic R. Baufeld C. Calcagno N. El Fatimy R. (2017). The TREM2-APOE pathway drives the transcriptional phenotype of dysfunctional microglia in neurodegenerative diseases. *Immunity* 47 566–581. 10.1016/j.immuni.2017.08.008 28930663 PMC5719893

[B50] Larochelle C. Cayrol R. Kebir H. Alvarez J. I. Lécuyer M. Ifergan I. (2012). Melanoma cell adhesion molecule identifies encephalitogenic T lymphocytes and promotes their recruitment to the central nervous system. *Brain* 135 2906–2924. 10.1093/brain/aws212 22975388

[B51] Leone M. A. Mandrioli J. Russo S. Cucovici A. Gianferrari G. Lisnic V. (2022). Neutrophils-to-Lymphocyte ratio is associated with progression and overall survival in amyotrophic lateral sclerosis. *Biomedicines* 10:354. 10.3390/biomedicines10020354 35203564 PMC8962424

[B52] Li M. Ransohoff R. M. (2008). Multiple roles of chemokine CXCL12 in the central nervous system: a migration from immunology to neurobiology. *Prog. Neurobiol.* 84 116–131. 10.1016/j.pneurobio.2007.11.003 18177992 PMC2324067

[B53] Li Q. Cheng Z. Zhou L. Darmanis S. Neff N. F. Okamoto J. (2019). Developmental heterogeneity of microglia and brain myeloid cells revealed by deep Single-Cell RNA sequencing. *Neuron* 101 207–223. 10.1016/j.neuron.2018.12.006 30606613 PMC6336504

[B54] Lin C. Y. Pfluger C. M. Henderson R. D. McCombe P. A. (2012). Reduced levels of interleukin 33 and increased levels of soluble ST2 in subjects with amyotrophic lateral sclerosis. *J. Neuroimmunol.* 249 93–95. 10.1016/j.jneuroim.2012.05.001 22633272

[B55] Liu E. Karpf L. Bohl D. (2021). Neuroinflammation in amyotrophic lateral sclerosis and frontotemporal dementia and the interest of induced pluripotent stem cells to study immune cells interactions with neurons. *Front. Mol. Neurosci*. 14:767041. 10.3389/fnmol.2021.767041 34970118 PMC8712677

[B56] Liu J. Prell T. Stubendorff B. Keiner S. Ringer T. Gunkel A. (2016). Down-regulation of purinergic P2X7 receptor expression and intracellular calcium dysregulation in peripheral blood mononuclear cells of patients with amyotrophic lateral sclerosis. *Neurosci. Lett.* 630 77–83. 10.1016/j.neulet.2016.07.039 27453058

[B57] Liu Z. Cheng X. Zhong S. Zhang X. Liu C. Liu F. (2020). Peripheral and central nervous system immune response crosstalk in amyotrophic lateral sclerosis. *Front. Neurosci.* 14:575. 10.3389/fnins.2020.00575 32612503 PMC7308438

[B58] Lochhead J. J. Yang J. Ronaldson P. T. Davis T. P. (2020). Structure, function, and regulation of the Blood-Brain barrier tight junction in central nervous system disorders. *Front. Physiol.* 11:914. 10.3389/fphys.2020.00914 32848858 PMC7424030

[B59] Lu C. Allen K. Oei F. Leoni E. Kuhle J. Tree T. (2016). Systemic inflammatory response and neuromuscular involvement in amyotrophic lateral sclerosis. *Neurol. Neuroimmunol. Neuroinflamm*. 3:e244. 10.1212/NXI.0000000000000244 27308305 PMC4897985

[B60] Marchetti L. Francisco D. Soldati S. Haghayegh J. N. Barcos S. Gruber I. (2022). ACKR1 favors transcellular over paracellular T-cell diapedesis across the blood-brain barrier in neuroinflammation in vitro. *Eur. J. Immunol.* 52 161–177. 10.1002/eji.202149238 34524684 PMC9293480

[B61] Martinez-Merino L. Iridoy M. Galbete A. Roldán M. Rivero A. Acha B. (2018). Evaluation of chitotriosidase and CC-Chemokine ligand 18 as biomarkers of microglia activation in amyotrophic lateral sclerosis. *Neurodegener. Dis.* 18 208–215. 10.1159/000490920 30134252

[B62] Masrori P. Beckers J. Gossye H. Van Damme P. (2022). The role of inflammation in neurodegeneration: novel insights into the role of the immune system in C9orf72 HRE-mediated ALS/FTD. *Mol. Neurodegener.* 17:22. 10.1186/s13024-022-00525-z 35303907 PMC8932121

[B63] Matsumoto J. Takata F. Machida T. Takahashi H. Soejima Y. Funakoshi M. (2014). Tumor necrosis factor-α-stimulated brain pericytes possess a unique cytokine and chemokine release profile and enhance microglial activation. *Neurosci. Lett.* 578 133–138. 10.1016/j.neulet.2014.06.052 24993300

[B64] Mattei D. Notter T. (2020). Basic concept of microglia biology and neuroinflammation in relation to psychiatry. *Curr. Top. Behav. Neurosci*. 44 9–34. 10.1007/7854_2018_83 30739307

[B65] Mattila O. S. Strbian D. Saksi J. Pikkarainen T. O. Rantanen V. Tatlisumak T. (2011). Cerebral mast cells mediate blood-brain barrier disruption in acute experimental ischemic stroke through perivascular gelatinase activation. *Stroke* 42 3600–3605. 10.1161/STROKEAHA.111.632224 21980200

[B66] McCombe P. A. Lee J. D. Woodruff T. M. Henderson R. D. (2020). The peripheral immune system and amyotrophic lateral sclerosis. *Front. Neurol.* 11:279. 10.3389/fneur.2020.00279 32373052 PMC7186478

[B67] Mcgill R. B. Steyn F. J. Ngo S. T. Thorpe K. A. Heggie S. Henderson R. D. (2021). Monocyte CD14 and HLA-DR expression increases with disease duration and severity in amyotrophic lateral sclerosis. *Amyotroph. Lateral Scler. Frontotemporal Degener.* [Online ahead of print] 10.1080/21678421.2021.1964531 34396845

[B68] Mehta P. Raymond J. Punjani R. Larson T. Han M. Bove F. (2022). Incidence of amyotrophic lateral sclerosis in the United States, 2014-2016. *Amyotroph. Lateral Scler. Frontotemporal Degener.* [Online ahead of print] 10.1080/21678421.2021.2023190 35023792

[B69] Mifflin L. Hu Z. Dufort C. Hession C. C. Walker A. J. Niu K. (2021). A RIPK1-regulated inflammatory microglial state in amyotrophic lateral sclerosis. *Proc. Natl. Acad. Sci*. 118:e2025102118. 10.1073/pnas.2025102118 33766915 PMC8020785

[B70] Miyazaki K. Ohta Y. Nagai M. Morimoto N. Kurata T. Takehisa Y. (2011). Disruption of neurovascular unit prior to motor neuron degeneration in amyotrophic lateral sclerosis. *J. Neurosci. Res.* 89 718–728. 10.1002/jnr.22594 21337372

[B71] Mohamed L. A. Markandaiah S. S. Bonanno S. Pasinelli P. Trotti D. (2019). Excess glutamate secreted from astrocytes drives upregulation of P-glycoprotein in endothelial cells in amyotrophic lateral sclerosis. *Exp. Neurol.* 316 27–38. 10.1016/j.expneurol.2019.04.002 30974102 PMC6506236

[B72] Murdock B. J. Zhou T. Kashlan S. R. Little R. J. Goutman S. A. Feldman E. L. (2017). Correlation of peripheral immunity with rapid amyotrophic lateral sclerosis progression. *JAMA Neurol.* 74:1446. 10.1001/jamaneurol.2017.2255 28973548 PMC5822195

[B73] Naor S. Keren Z. Bronshtein T. Goren E. Machluf M. Melamed D. (2009). Development of ALS-like disease in SOD-1 mice deficient of B lymphocytes. *J. Neurol.* 256 1228–1235. 10.1007/s00415-009-5097-3 19280101

[B74] Nardo G. Trolese M. C. de Vito G. Cecchi R. Riva N. Dina G. (2016). Immune response in peripheral axons delays disease progression in SOD1G93A mice. *J. Neuroinflamm.* 13:261 10.1186/s12974-016-0732-2 27717377 PMC5055725

[B75] Nguyen B. Bix G. Yao Y. (2021). Basal lamina changes in neurodegenerative disorders. *Mol. Neurodegener.* 16:81. 10.1186/s13024-021-00502-y 34876200 PMC8650282

[B76] Nishihara H. Soldati S. Mossu A. Rosito M. Rudolph H. Muller W. A. (2020). Human CD4+ T cell subsets differ in their abilities to cross endothelial and epithelial brain barriers in vitro. *Fluids Barriers CNS* 17:3. 10.1186/s12987-019-0165-2 32008573 PMC6996191

[B77] Noh M. Y. Cho K. A. Kim H. Kim S. Kim S. H. (2014). Erythropoietin modulates the immune-inflammatory response of a SOD1G93A transgenic mouse model of amyotrophic lateral sclerosis (ALS). *Neurosci. Lett.* 574 53–58. 10.1016/j.neulet.2014.05.001 24820540

[B78] Ouali A. N. Schurr C. Olde H. F. Tang L. Li Q. Tasdogan A. (2018). NF-kappaB activation in astrocytes drives a stage-specific beneficial neuroimmunological response in ALS. *EMBO J.* 10.15252/embj.201798697 29875132 PMC6092622

[B79] Pan Y. Nicolazzo J. A. (2022). Altered blood–brain barrier and blood–spinal cord barrier dynamics in amyotrophic lateral sclerosis: impact on medication efficacy and safety. *Br. J. Pharmacol.* 10.1111/bph.15802 [Epub ahead of print]. 35048358

[B80] Peake K. Manning J. Lewis C. Tran K. Rossi F. Krieger C. (2017). Bone Marrow-Derived cell accumulation in the spinal cord is independent of peripheral mobilization in a mouse model of amyotrophic lateral sclerosis. *Front. Neurol.* 8:75. 10.3389/fneur.2017.00075 28337172 PMC5340765

[B81] Pennati A. Asress S. Glass J. D. Galipeau J. (2018). Adoptive transfer of IL-10+ regulatory B cells decreases myeloid-derived macrophages in the central nervous system in a transgenic amyotrophic lateral sclerosis model. *Cell. Mol. Immunol.* 15 727–730. 10.1038/cmi.2017.152 29307886 PMC6123431

[B82] Perner C. Perner F. Stubendorff B. Förster M. Witte O. W. Heidel F. H. (2018). Dysregulation of chemokine receptor expression and function in leukocytes from ALS patients. *J. Neuroinflamm.* 15:99. 10.1186/s12974-018-1135-3 29592817 PMC5874995

[B83] Pieper C. Pieloch P. Galla H. (2013). Pericytes support neutrophil transmigration via interleukin-8 across a porcine co-culture model of the blood–brain barrier. *Brain Res.* 1524 1–11. 10.1016/j.brainres.2013.05.047 23769734

[B84] Pronto-Laborinho A. Pinto S. Gromicho M. Pereira M. Swash M. de Carvalho M. (2019). Interleukin-6 and amyotrophic lateral sclerosis. *J. Neurol. Sci.* 398 50–53. 10.1016/j.jns.2019.01.026 30682521

[B85] Qin X. Wang J. Chen S. Liu G. Wu C. Lv Q. (2022). Astrocytic p75NTR expression provoked by ischemic stroke exacerbates the blood–brain barrier disruption. *Glia* 70 892–912. 10.1002/glia.24146 35064700

[B86] Qosa H. Lichter J. Sarlo M. Markandaiah S. S. McAvoy K. Richard J. (2016). Astrocytes drive upregulation of the multidrug resistance transporter ABCB1 (P-Glycoprotein) in endothelial cells of the blood-brain barrier in mutant superoxide dismutase 1-linked amyotrophic lateral sclerosis. *Glia* 64 1298–1313. 10.1002/glia.23003 27158936 PMC5541958

[B87] Rabinovich-Nikitin I. Ezra A. Barbiro B. Rabinovich-Toidman P. Solomon B. (2016). Chronic administration of AMD3100 increases survival and alleviates pathology in SOD1G93A mice model of ALS. *J. Neuroinflamm.* 13:123. 10.1186/s12974-016-0587-6 27230771 PMC4882847

[B88] Rolfes L. Schulte-Mecklenbeck A. Schreiber S. Vielhaber S. Herty M. Marten A. (2021). Amyotrophic lateral sclerosis patients show increased peripheral and intrathecal T-cell activation. *Brain Commun.* 3:fcab157. 10.1093/braincomms/fcab157 34405141 PMC8363480

[B89] Rusconi M. Gerardi F. Santus W. Lizio A. Sansone V. A. Lunetta C. (2017). Inflammatory role of dendritic cells in Amyotrophic Lateral Sclerosis revealed by an analysis of patients’ peripheral blood. *Sci. Rep.* 7:7853. 10.1038/s41598-017-08233-1 28798369 PMC5552769

[B90] Salamone P. Fuda G. Casale F. Marrali G. Lunetta C. Caponnetto C. (2020). G-CSF (filgrastim) treatment for amyotrophic lateral sclerosis: protocol for a phase II randomised, double-blind, placebo-controlled, parallel group, multicentre clinical study (STEMALS-II trial). *BMJ Open* 10:e34049. 10.1136/bmjopen-2019-034049 32209625 PMC7202695

[B91] Sasaki S. (2015). Alterations of the blood-spinal cord barrier in sporadic amyotrophic lateral sclerosis. *Neuropathology* 35 518–528. 10.1111/neup.12221 26242689

[B92] Scott A. (2017). Drug therapy: on the treatment trail for ALS. *Nature* 550 S120–S121. 10.1038/550S120a 29045376

[B93] Shefner J. Heiman Patterson T. Pioro E. P. Wiedau Pazos M. Liu S. Zhang J. (2020). Long-term edaravone efficacy in amyotrophic lateral sclerosis: post-hoc analyses of Study 19 (MCI186-19). *Muscle Nerve*. 61 218–221. 10.1002/mus.26740 31621933 PMC7004197

[B94] Shi N. Kawano Y. Tateishi T. Kikuchi H. Osoegawa M. Ohyagi Y. (2007). Increased IL-13-producing T cells in ALS: positive correlations with disease severity and progression rate. *J. Neuroimmunol.* 182 232–235. 10.1016/j.jneuroim.2006.10.001 17097743

[B95] Shiraishi W. Yamasaki R. Hashimoto Y. Ko S. Kobayakawa Y. Isobe N. (2021). Clearance of peripheral nerve misfolded mutant protein by infiltrated macrophages correlates with motor neuron disease progression. *Sci. Rep*. 11:16438. 10.1038/s41598-021-96064-6 34385589 PMC8360983

[B96] Signorile A. Ferretta A. Ruggieri M. Paolicelli D. Lattanzio P. Trojano M. (2021). Mitochondria, oxidative stress, cAMP signalling and apoptosis: a crossroads in lymphocytes of multiple sclerosis, a possible role of nutraceutics. *Antioxidants*. 10:21. 10.3390/antiox10010021 33379309 PMC7823468

[B97] Song J. Wu C. Korpos E. Zhang X. Agrawal S. M. Wang Y. (2015). Focal MMP-2 and MMP-9 activity at the Blood-Brain barrier promotes Chemokine-Induced leukocyte migration. *Cell Rep.* 10 1040–1054. 10.1016/j.celrep.2015.01.037 25704809

[B98] Spiller K. J. Khan T. Dominique M. A. Restrepo C. R. Cotton-Samuel D. Levitan M. (2019). Reduction of matrix metalloproteinase 9 (MMP-9) protects motor neurons from TDP-43-triggered death in rNLS8 mice. *Neurobiol. Dis.* 124 133–140. 10.1016/j.nbd.2018.11.013 30458231 PMC7053168

[B99] Strickland M. R. Ibanez K. R. Yaroshenko M. Diaz C. C. Borchelt D. R. Chakrabarty P. (2020). IL-10 based immunomodulation initiated at birth extends lifespan in a familial mouse model of amyotrophic lateral sclerosis. *Sci. Rep.* 10:20862. 10.1038/s41598-020-77564-3 33257786 PMC7705692

[B100] Sun Q. Huo Y. Bai J. Wang H. Wang H. Yang F. (2022). Inflammatory cytokine levels in patients with sporadic amyotrophic lateral sclerosis. *Neurodegener. Dis.* 21 87–92. 10.1159/000522078 35124669

[B101] Sunnemark D. Eltayeb S. Nilsson M. Wallstrom E. Lassmann H. Olsson T. (2005). CX3CL1 (fractalkine) and CX3CR1 expression in myelin oligodendrocyte glycoprotein-induced experimental autoimmune encephalomyelitis: kinetics and cellular origin. *J. Neuroinflammation*. 2:17. 10.1186/1742-2094-2-17 16053521 PMC1188067

[B102] Sweeney M. D. Zhao Z. Montagne A. Nelson A. R. Zlokovic B. V. (2019). Blood-Brain barrier: from physiology to disease and back. *Physiol. Rev.* 99 21–78. 10.1152/physrev.00050.2017 30280653 PMC6335099

[B103] Takata F. Nakagawa S. Matsumoto J. Dohgu S. (2021). Blood-Brain barrier dysfunction amplifies the development of neuroinflammation: understanding of cellular events in brain microvascular endothelial cells for prevention and treatment of BBB dysfunction. *Front. Cell. Neurosci.* 15:661838. 10.3389/fncel.2021.661838 34588955 PMC8475767

[B104] Theoharides T. C. Tsilioni I. (2020). Amyotrophic lateral sclerosis, neuroinflammation, and cromolyn. *Clin. Ther.* 42 546–549. 10.1016/j.clinthera.2020.01.010 32044139

[B105] Tortarolo M. Lo Coco D. Veglianese P. Vallarola A. Giordana M. T. Marcon G. (2017). Amyotrophic lateral sclerosis, a multisystem pathology: insights into the role of TNFα. *Mediat. Inflamm.* 2017:2985051. 10.1155/2017/2985051 29081600 PMC5610855

[B106] Tortelli R. Zecca C. Piccininni M. Benmahamed S. Dell’Abate M. T. Barulli M. R. (2020). Plasma inflammatory cytokines are elevated in ALS. *Front. Neurol.* 11:552295. 10.3389/fneur.2020.552295 33281700 PMC7691268

[B107] Trias E. King P. H. Si Y. Kwon Y. Varela V. Ibarburu S. (2018). Mast cells and neutrophils mediate peripheral motor pathway degeneration in ALS. *JCI Insight* 3:e123249. 10.1172/jci.insight.123249 30282815 PMC6237484

[B108] Trias E. Kovacs M. King P. H. Si Y. Kwon Y. Varela V. (2020). Schwann cells orchestrate peripheral nerve inflammation through the expression of CSF1, IL-34, and SCF in amyotrophic lateral sclerosis. *Glia* 68 1165–1181. 10.1002/glia.23768 31859421 PMC7269115

[B109] Trolese M. C. Mariani A. Terao M. de Paola M. Fabbrizio P. Sironi F. (2020). CXCL13/CXCR5 signalling is pivotal to preserve motor neurons in amyotrophic lateral sclerosis. *EBioMedicine* 62:103097. 10.1016/j.ebiom.2020.103097 33161233 PMC7670099

[B110] van Vliet E. A. Iyer A. M. Mesarosova L. Çolakoglu H. Anink J. J. van Tellingen O. (2020). Expression and cellular distribution of P-Glycoprotein and breast cancer resistance protein in amyotrophic lateral sclerosis patients. *J. Neuropathol. Exp. Neurol*. 79 266–276. 10.1093/jnen/nlz142 31999342 PMC7036662

[B111] Vandoorne T. De Bock K. Van Den Bosch L. (2018). Energy metabolism in ALS: an underappreciated opportunity? *Acta Neuropathol.* 135 489–509. 10.1007/s00401-018-1835-x 29549424 PMC5978930

[B112] Wang Y. Jin S. Sonobe Y. Cheng Y. Horiuchi H. Parajuli B. (2014). Interleukin-1beta induces blood-brain barrier disruption by downregulating Sonic hedgehog in astrocytes. *PLoS One* 9:e110024. 10.1371/journal.pone.0110024 25313834 PMC4196962

[B113] Waters S. Swanson M. E. V. Dieriks B. V. Zhang Y. B. Grimsey N. L. Murray H. C. (2021). Blood-spinal cord barrier leakage is independent of motor neuron pathology in ALS. *Acta Neuropathol. Commun.* 9:144. 10.1186/s40478-021-01244-0 34446086 PMC8393479

[B114] Wei Q. Zhou Q. Chen Y. Ou R. Cao B. Xu Y. (2017). Analysis of SOD1 mutations in a Chinese population with amyotrophic lateral sclerosis: a case-control study and literature review. *Sci. Rep.* 7:44606. 10.1038/srep44606 28291249 PMC5349524

[B115] Winkler E. A. Sengillo J. D. Sullivan J. S. Henkel J. S. Appel S. H. Zlokovic B. V. (2013). Blood–spinal cord barrier breakdown and pericyte reductions in amyotrophic lateral sclerosis. *Acta Neuropathol.* 125 111–120. 10.1007/s00401-012-1039-8 22941226 PMC3535352

[B116] Wosiski-Kuhn M. Robinson M. Strupe J. Arounleut P. Martin M. Caress J. (2019). IL6 receptor358 Ala variant and trans-signaling are disease modifiers in amyotrophic lateral sclerosis. *Neurol. Neuroimmunol. Neuroinflamm*. 6:e631. 10.1212/NXI.0000000000000631 31611269 PMC6865852

[B117] Wu D. Fu X. Zhang Y. Li Q. Ye L. Han S. (2020). The protective effects of C16 peptide and angiopoietin-1 compound in lipopolysaccharide-induced acute respiratory distress syndrome. *Exp. Biol. Med.* 245 1683–1696. 10.1177/1535370220953791 32915636 PMC7802387

[B118] Wu Y. Yang X. Li X. Wang H. Wang T. (2020). Elevated cerebrospinal fluid homocysteine is associated with blood-brain barrier disruption in amyotrophic lateral sclerosis patients. *Neurol. Sci.* 41 1865–1872. 10.1007/s10072-020-04292-x 32086685

[B119] Xu L. Chen L. Wang S. Feng J. Liu L. Liu G. (2020). Incidence and prevalence of amyotrophic lateral sclerosis in urban China: a national population-based study. *J. Neurol. Neurosurg. Psychiatry* 91 520–525. 10.1136/jnnp-2019-322317 32139654

[B120] Yamadera M. Fujimura H. Inoue K. Toyooka K. Mori C. Hirano H. (2015). Microvascular disturbance with decreased pericyte coverage is prominent in the ventral horn of patients with amyotrophic lateral sclerosis. *Amyotroph. Lateral Scler. Frontotemporal Degener*. 16 393–401. 10.3109/21678421.2015.1011663 25740316

[B121] Yu X. Ji C. Shao A. (2020). Neurovascular unit dysfunction and neurodegenerative disorders. *Front. Neurosci.* 14:334. 10.3389/fnins.2020.00334 32410936 PMC7201055

[B122] Yuan J. Amin P. Ofengeim D. (2019). Necroptosis and RIPK1-mediated neuroinflammation in CNS diseases. *Nat. Rev. Neurosci.* 20 19–33. 10.1038/s41583-018-0093-1 30467385 PMC6342007

[B123] Zhang J. Liu Y. Liu X. Li S. Cheng C. Chen S. (2018). Dynamic changes of CX3CL1/CX3CR1 axis during microglial activation and motor neuron loss in the spinal cord of ALS mouse model. *Transl. Neurodegener.* 7:35. 10.1186/s40035-018-0138-4 30607245 PMC6309063

[B124] Zhao W. Beers D. R. Hooten K. G. Sieglaff D. H. Zhang A. Kalyana-Sundaram S. (2017). Characterization of gene expression phenotype in amyotrophic lateral sclerosis monocytes. *JAMA Neurol.* 74:677. 10.1001/jamaneurol.2017.0357 28437540 PMC5822209

[B125] Zhao W. Beers D. R. Liao B. Henkel J. S. Appel S. H. (2012). Regulatory T lymphocytes from ALS mice suppress microglia and effector T lymphocytes through different cytokine-mediated mechanisms. *Neurobiol. Dis.* 48 418–428. 10.1016/j.nbd.2012.07.008 22820142 PMC3897268

[B126] Zhao X. Yang F. Wang H. Cui F. Li M. Sun B. (2020). The increase in CSF total protein and immunoglobulins in Chinese patients with sporadic amyotrophic lateral sclerosis: a retrospective study. *J. Neurol. Sci.* 414:116840. 10.1016/j.jns.2020.116840 32388062

